# High‐resolution mapping of *Yr78*, an adult plant resistance gene to wheat stripe rust

**DOI:** 10.1002/tpg2.20212

**Published:** 2022-04-25

**Authors:** Chen Dang, Junli Zhang, Jorge Dubcovsky

**Affiliations:** ^1^ Dep. of Plant Sciences Univ. of California Davis CA 95616 USA; ^2^ Howard Hughes Medical Institute Chevy Chase MD 20815 USA

## Abstract

Wheat stripe rust, caused by *Puccinia striiformis* f. sp. *tritici* (*Pst*), is responsible for significant yield losses worldwide, which can be minimized by the deployment of *Pst* resistance genes. *Yr78* is an adult plant partial‐resistance gene that has remained effective against the post‐2000 virulent *Pst* races. In this study, we generated a high‐resolution map of *Yr78* based on 6,124 segregating chromosomes. We mapped *Yr78* within a 0.05‐cM interval on the short arm of chromosome 6B, which corresponds to an 11.16 Mb region between *TraesCS6B02G116200* and *TraesCS6B02G118000* in the ‘Chinese Spring’ Ref Seq. v1.1 genome. This interval is likely larger because it includes the unassembled *NOR‐B2* region, which may have contributed to the low recombination rate detected in this region. The *Yr78* candidate region includes 15 genes that were prioritized for future functional studies based on their annotated function and polymorphisms between susceptible and resistant genotypes. Using exome capture data, we identified five major haplotypes in the candidate gene region, with the H1 haplotype associated with *Yr78*. The H1 haplotype was not detected in tetraploid wheat (*Triticum turgidum* L.) but was found in ∼30% of the common wheat cultivars (*Triticum aestivum* L.), suggesting that the associated resistance to stripe rust may have favored the selection of this haplotype. We developed two diagnostic molecular markers for the H1 haplotype that will facilitate the deployment of *Yr78* in wheat breeding programs.

## Core Ideas

- *Yr78* is tightly linked to the *NOR‐B2* locus on a chromosome 6B region of limited recombination.
- *Yr78* was mapped to a 0.05‐cM region that includes 15 high‐confidence annotated genes.
- There is a large gap in the assembly of the *NOR‐B2* region in currently published genomes.
- The H1 haplotype is the only one associated with a resistant allele of *Yr78*.
- We developed two diagnostic markers to accelerate the deployment of *Yr78*

**Abbreviations**
CC: coiled coil
CS: ‘Chinese Spring’
IT: infection type
KASP: Kompetitive Allele‐Specific Polymerase chain reaction
NBS‐LRR: nucleotide‐binding site, leucine‐rich repeat
NOR: nucleolar organizing region
Pst: *Puccinia striiformis* f. sp. *tritici*
QTL: quantitative trait locus/loci
rDNA: ribosomal DNA
S–TPK: serine–threonine protein kinase
SEV: severity
SNP: single‐nucleotide polymorphism

## 1 INTRODUCTION

Wheat (*Triticum ssp*.) is an important crop for global food security, with more than 750 Tg produced each year (FAOSTAT). Continuous increases in wheat production are required to feed a growing human population, and therefore, it is important to minimize losses caused by pathogens. Wheat stripe rust, caused by *Puccinia striiformis* f. sp. *tritici* (*Pst*), is currently one of the most severe diseases of wheat, affecting most wheat‐growing countries (Chen, 2020). Although fungicide applications can be used to reduce stripe rust epidemics, this is not economically feasible in all countries and can be detrimental to the environment and human health if not properly used.

The appearance of more virulent and aggressive *Pst* races at the beginning of this century (Hovmøller et al., 2010; Milus et al., 2008) has resulted in the loss of many of the previously effective *Pst* resistance genes (*Yr*) and in large yield losses worldwide (Chen, 2020; Wellings, 2011). The new *Pst* races have shorter latent periods and faster spore production under warmer temperatures than previous races (Hovmøller et al., 2016; Markell & Milus, 2008; Milus et al., 2008), which help them to extend the infection season and to spread to warmer regions of the world. In susceptible cultivars not treated with fungicides, these new *Pst* races can cause severe yield losses (Chen, 2005).

The spread of the new races after the year 2000, together with the appearance of virulent combinations from the old races, resulted in rapid erosion of many of the resistance genes used extensively in the past including *Yr8*, *Yr9*, *Yr17*, *Yr24*, and *Yr27* (Wan & Chen, 2014). A few major *Pst* resistance genes, such as *Yr5* (Marchal et al., 2018) and *Yr15* (Klymiuk et al., 2018), remained effective against the post‐2000 *Pst* virulent races (Wan & Chen, 2014). However, rare virulent races have been reported for both genes (Hovmoller & Justesen, 2007; Wellings & Mcintosh, 1990; Zhan et al., 2016), highlighting the risk of relying on a few resistance genes.

*Pst* resistance genes are usually classified into race‐specific resistance (also known as seedling resistance) and race nonspecific resistance (also known as partial resistance or adult plant resistance). Race‐specific resistance genes typically encode nucleotide‐binding site, leucine‐rich repeat (NBS‐LRR) proteins, which can directly or indirectly detect pathogen effectors and trigger plant defense responses. However, the pathogens can break this type of resistance by simply modifying or losing effectors to avoid detection (DeYoung & Innes, 2006; Jones & Dangl, 2006). This has resulted in a history of pathogens overcoming the resistance conferred by major resistance genes soon after their deployment in agriculture and has led to the search for more durable forms of resistance (Mundt, 2014).

Adult plant resistance genes, also known as partial‐resistance genes, are usually race nonspecific and have been historically more durable than race‐specific resistance genes. In contrast to race‐specific resistance genes against *Pst*, race nonspecific genes are diverse, with the cloned genes including an ABC transporter (Krattinger et al., 2009), a kinase ‐START domain protein (Fu et al., 2009), a hexose transporter (Moore et al., 2015), and putative kinase‐pseudokinase protein (Klymiuk et al., 2018). Identifying, mapping, cloning, and deploying new partial‐resistance genes effective against the new *Pst* races is an important priority to defeat current *Pst* races and avoid a new pandemic (Lowe et al., 2011).

Previously, our lab conducted a genome‐wide association study in six different environments from the year 2011 to 2013 (Maccaferri et al., 2015) and discovered a strong quantitative trail locus (QTL) for *Pst* adult plant resistance on chromosome arm 6BS in a region where no previously named *Yr*gene has been mapped (Dong et al., 2017). This gene, designated *Yr78*, was validated in 10 biparent populations and explained up to 45.9% of the variation in infection type (IT). *Yr78* was mapped within a 4.5‐cM region delimited by markers IWA7257 and IWA4408 in the population PI 519805 × ‘Avocet’ ‘S’ (Dong et al., 2017). Since we first evaluated it in 2011, *Yr78* has remained effective and stable under heavy *Pst* pressure for 10 years. On the basis of its stability and value for wheat breeding, we developed a high‐density map with the long‐term goal of cloning this gene. In this study, we mapped *Yr78* to a 0.05‐cM (11.16 Mb) region including 15 high‐confidence genes. We discuss the potential of these genes as candidates for *Yr78* and provide tightly linked markers to facilitate its deployment in wheat breeding programs.

**Core Ideas**

- *Yr78* is tightly linked to the *NOR‐B2* locus on a chromosome 6B region of limited recombination.
- *Yr78* was mapped to a 0.05‐cM region that includes 15 high‐confidence annotated genes.
- There is a large gap in the assembly of the *NOR‐B2*region in currently published genomes.
- The H1 haplotype is the only one associated with a resistant allele of *Yr78*.
- We developed two diagnostic markers to accelerate the deployment of *Yr78*.

## 2 MATERIALS AND METHODS

### 2.1 Development of the high‐density mapping population

The PI 519805 × Avocet ‘S’ biparental population used for the initial mapping of *Yr78* also segregated for a separate *Pst* resistance QTL on chromosome arm 1BL designated as *QYr.ucw‐1B* (Dong et al., 2017). To minimize variability and visualize better the effect of *Yr78*, we developed a mapping population fixed for the *QYr.ucw‐1B* susceptible allele from Avocet ‘S’ and segregating for *Yr78*. Using the peak single‐nucleotide polymorphism (SNP) marker for *QYr.ucw‐1B* (IWA802) and *Yr78* flanking markers IWA7257 and IWA4408 we selected 25 F_2_ plants segregating only for *Yr78*. The progeny of these plants was used to develop the high‐density mapping population.

### 2.2 Field evaluations for resistance

Field trials to evaluate the recombinant families were performed between 2018 and 2019 at the University of California Experimental Field Station in Davis, CA (38°31′33″ N, 121°46′30″ W, elevation 16 m) designated hereafter as UC Davis. Several families with critical recombination events were planted again in 2020–2021 for validation. We planted the experiments in mid‐November and included a susceptible border around the tested lines (D6301). We inoculated the susceptible border in February with *Pst* spores from a mixture of races collected from the previous field season at UC Davis. A list of the *Pst* races present in the UC Davis fields in 2015 and 2016 and their virulences was published previously (Dong et al., 2017). In 2018, seven *Pst* samples collected at UC Davis revealed the presence of races PSTv‐4, PSTv‐37, PSTv‐202, and PSTv‐220. The same races, with the addition of PSTv‐47 and PSTv‐52, were detected in a survey of 27 additional samples collected in other parts of California between 2018 and 2020. All *Pst* race determinations were performed by Dr. Xianming Chen at Washington State University.

For inoculation, spores were imbedded in talcum powder to ensure uniform disease pressure. For each of the F_3_ plants carrying recombination events between the flanking markers, we performed progeny tests in the field using an average of 16.4 F_4_ plants. Individual plants were sown in 1‐m rows, including six plants per row with a separation of 30 cm between rows to facilitate disease evaluation. Plants were evaluated for IT and severity (SEV) three times starting when 50% of the lines were heading and ending when all the plants had headed. The statistical analyses were based on the flag leaf data collected at the highest infection point. Infection type was recorded using the McNeal's 0 (resistant)–9 (susceptible) scale (Line & Qayoum, 1992). The extreme scores 0 and 9 were not observed in our population, so our IT scores range from 1 to 8. Disease severity indicates the percentage of the flag leaf covered with pustules.

### 2.3 Exome captures of parental lines and development of SNP markers

Exome captures of Avocet ‘S’ and PI 519805 were conducted using the assay developed by NimbleGen (Krasileva et al., 2017). Captured libraries were sequenced using the Illumina platform with 150‐bp paired‐end reads at the UC Davis Genome Center. The quality of sequencing reads was checked with software FastQC v0.11.5 (https://www.bioinformatics.babraham.ac.uk/projects/fastqc/). Adapters and low‐quality bases were trimmed with the programs scythe v0.991 (https://github.com/vsbuffalo/scythe) and sickle v1.33 (https://github.com/vsbuffalo/scythe). Trimmed reads were aligned to the ‘Chinese Spring’ (CS) RefSeq v1.1 genome assembly using bwa v0.7.9a (*bwa aln* and *bwa sampe*) (Li & Durbin, 2009). Alignments were sorted using Samtools v0.1.19 (Li et al., 2009), and duplicate reads were marked with Picard tools v2.8.1 (http://broadinstitute.github.io/picard/). Variants were called with GATK v4.0.4.0 (https://github.com/broadinstitute/gatk/). The SNP effects were annotated using SnpEff v4.3t (Cingolani et al., 2012) on the RefSeq v1.1 annotation (International Wheat Genome Sequencing Consortium, 2018). Statistics for the mapped reads were obtained from the bam files with command ‘samtools flagstat’.

### 2.4 Haplotype analysis

To improve the markers for *Yr78*, we performed a haplotype analysis of the candidate gene region. We compared the exome capture results of Avocet ‘S’ and PI 519805 with exome capture data from other 55 common wheat lines extracted from the T3/Wheat database project 2017_WheatCAP_UCD (https://wheat.triticeaetoolbox.org/search/genotyping_data_projects). Diploid einkorn (*T. monococcum* L.) and Tausch's goat grass (*Aegilops tauschii* Coss.) accessions and tetraploid wild emmer [*T. turgidum* L. subsp. *dicoccoides* (Körn. ex Asch. & Graebn.) Thell.] (genotype Zavitan) were not included. Lines and SNPs with >30% missing data were removed, and heterozygous calls were replaced by missing data for the cluster analysis, which was performed using the R functions ‘dist’ and ‘hclust’ (method = ‘ward.D2’) (R Core Team, 2021).

For the SNPs selected to differentiate the haplotype carrying *Yr78*, we developed Kompetitive Allele‐Specific Polymerase chain reaction (KASP) markers and validated them in a biparental population derived from the cross ‘Berkut’ × RAC875 (Zhang et al., 2018). This population was evaluated for resistance to stripe rust at UC Davis in the 2015–2016 field season. The KASP markers were also validated in a set of 10 lines previously confirmed to carry a stripe rust resistance gene linked to marker IWA7257 (Dong et al., 2017) and a set of 22 highly susceptible lines selected from a previous study (Maccaferri et al., 2015). Finally, we tested the two markers that differentiate the *Yr78* resistant haplotype in 88 wild emmer, 92 emmer wheat [*T. turgidum* L. subsp. *dicoccon* (Schrank) Thell.], and 393 durum wheat [*T. turgidum* L. subsp. *durum* (Desf.) Husn.].

### 2.5 Genomic analyses

Genome sequences were obtained mainly from the CS RefSeq v1.1. (International Wheat Genome Sequencing Consortium, 2018) and from the genomes sequenced in the wheat PanGenome project (Walkowiak et al., 2020). The ‘Kronos’ EI v1 genome data was obtained from the Grassroots Data Repository at the Earlham Institute (https://opendata.earlham.ac.uk/opendata/data/Triticum_turgidum/EI/v1/). The ‘Fielder’ genome data was obtained from Sato et al. (2021) and the ‘Svevo’ genome from Maccaferri et al. (2019). Data for the assay for transposase‐accessible chromatin sequencing data was obtained from Lu et al. (2020).

## 3 RESULTS

### 3.1 Development of the *Yr78* high‐density mapping population and *Pst* evaluation

We obtained 3,062 F_3_ plants from 25 selected F_2_ plants homozygous for the susceptible *QYr.ucw‐1B* allele and segregating for *Yr78* flanking markers. Plants were grown in different batches in the greenhouse for DNA extraction and genotyping with *Yr78* flanking markers IWA7257 (RefSeq v1.1: 6BS 92,462,100) and IWA4408 (RefSeq v1.1: 6BS 119,978,760). We identified 159 F_3_ plants with recombination events between these two flanking markers resulting in a genetic distance of 2.6 cM between IWA7257 and IWA4408. On the basis of the physical distance between these two flanking markers (27.52 Mb), we estimated that the average ratio between physical and genetic distances in this region was 10.6 Mb cM^−1^.

**FIGURE 1:**
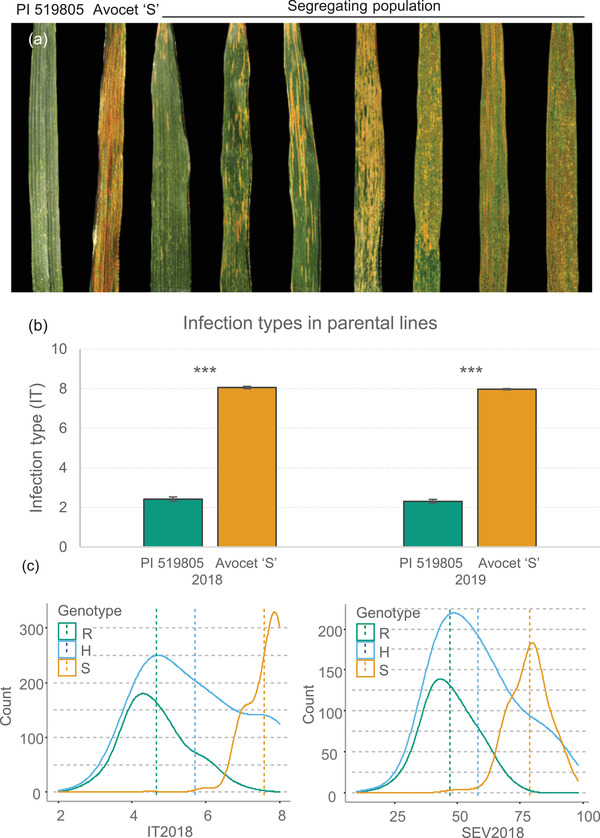
*Yr78* infection type (IT) and severity (SEV). (a) Reactions to field infection of *Puccinia striiformis* f. sp. *tritici* (*Pst*) in the resistant parental line PI 519805, susceptible parental line Avocet ‘S’, and seven segregating lines showing various reaction phenotypes. (b) Average IT values of Avocet ‘S’ and PI 519805 in 2018 and 2019 field experiments. Error bars are standard error of the means based on 18 plants (Supplemental Table S1). (c) Frequency distribution of plants homozygous resistant (R), homozygous susceptible (S) and heterozygous (H) for *Yr78* as determined by progeny tests of 105 recombinant families (1,721 plants, Supplemental Table S2). Note the closer position of the IT and SEV means of the heterozygous plants (blue dotted line) relative to the means of the resistant plants (green dotted line) than to the means of the susceptive plants (orange dotted line). This result indicates partial dominance of the *Yr78* resistant allele

To determine the *Yr78* allele in each of the 159 F_3_ plants with informative recombination events, we performed field progeny tests including an average of 16.4 F_4_ plants per recombinant family. In 2018, we evaluated 1,721 plants from 105 families, and the following year, 880 plants from 54 families (Figure 1a). Infections with *Pst* were strong and uniform both years, with all susceptible borders reaching IT scores of 8 and SEV > 80%. In both years, the average IT of the resistant parental line PI 519805 varied from 2.0 to 3.0, whereas the average IT of Avocet ‘S’ varied from 7.5 to 8.0, and the differences were highly significant both years (*P* < .001) (Figure 1b). In addition to *Yr78*, parental line PI 519805 carries the minor stripe rust resistant QTL *QYr.ucw‐1B*, which results in lower average IT scores than in resistant families from the population segregating only for *Yr78* (average IT values between 3.5 and 5.5; Table 1).

**TABLE 1:** Critical recombination events flanking *Yr78*

		Critical recombinants																					
Markers	RefSeq v1.1	R4	R10	R14	R21	R49	R50	R52	R58	R69	R113	R114	R128	R130	R134	R142	R147	R148	R162	R149	R154	R155	R169
	bp																						
CDM53	93,448,500	*R*	*R*	*S*	*H*	*R*	*S*	*R*	*R*	*S*	*H*	*R*	*H*	*H*	*R*	*R*	*S*	*S*	*H*	*H*	*H*	*S*	*R*
CDM88	101,735,482	*R*	*R*	*H*	*H*	*R*	*S*	*H*	*R*	*H*	*R*	*H*	*H*	*H*	*H*	*R*	*H*	*H*	*H*	*R*	*S*	*H*	*R*
CDM92	103,669,865	*R*	*R*	*H*	*H*	*R*	*S*	*H*	*R*	*H*	*R*	*H*	*H*	*H*	*H*	*R*	*H*	*H*	*S*	*R*	*S*	*H*	*R*
CDM95	106,474,975	*R*	*R*	*H*	*H*	*R*	*S*	*H*	*R*	*H*	*R*	*H*	*H*	*H*	*H*	*R*	*H*	*H*	*S*	*R*	*S*	*H*	*R*
CDM9‐1	107,773,849	*R*	*R*	*H*	*H*	*R*	*S*	*H*	*R*	*H*	*R*	*H*	*H*	*H*	*H*	*R*	*H*	*H*	*S*	*R*	*S*	*H*	*R*
CDM101	109,143,122	*R*	*R*	*H*	*H*	*R*	*S*	*H*	*R*	*H*	*R*	*H*	*H*	*H*	*H*	*R*	*H*	*H*	*S*	*R*	*S*	*H*	*R*
CDM102	109,143,155	*R*	*R*	*H*	*H*	*R*	*S*	*H*	*R*	*H*	*R*	*H*	*H*	*H*	*H*	*R*	*H*	*H*	*S*	*R*	*S*	*H*	*R*
Phenotype	–	*R*	*R*	*H*	*H*	*R*	*S*	*H*	*R*	*H*	*R*	*H*	*H*	*H*	*H*	*R*	*H*	*H*	*S*	*R*	*S*	*H*	*R*
CDM103	112,897,900	*R*	*R*	*H*	*H*	*H*	*S*	*H*	*R*	*H*	*R*	*H*	*H*	*H*	*H*	*H*	*H*	*H*	*S*	*R*	*S*	*H*	*R*
CDM13‐2	112,898,100	*R*	*R*	*H*	*H*	*H*	*S*	*H*	*R*	*H*	*R*	*H*	*H*	*H*	*H*	*H*	*H*	*H*	*S*	*R*	*S*	*H*	*R*
CDM15	112,905,564	*H*	*H*	*H*	*S*	*H*	*H*	*H*	*H*	*H*	*R*	*H*	*R*	*R*	*H*	*H*	*H*	*H*	*S*	*R*	*S*	*H*	*H*
CDM108	113,574,857	*H*	*H*	*H*	*S*	*H*	*H*	*H*	*H*	*H*	*R*	*H*	*R*	*R*	*H*	*H*	*H*	*H*	*S*	*R*	*S*	*H*	*H*
CDM109	114,260,360	*H*	*H*	*H*	*S*	*H*	*H*	*H*	*H*	*H*	*R*	*H*	*R*	*R*	*H*	*H*	*H*	*H*	*S*	*R*	*S*	*H*	*H*
CDM119	115,683,947	*H*	*H*	*H*	*S*	*H*	*H*	*H*	*H*	*H*	*R*	*H*	*R*	*R*	*H*	*H*	*H*	*H*	*S*	*R*	*S*	*H*	*H*
2018–2019 (2020–2021)	*N*	18 (18)	18 (13)	16	16 (15)	15 (15)	16 (15)	16	16 (13)	16	16	18	15 (31)	16 (32)	15	17 (13)	16	18	15	15	15	18	12 (11)
	*R*	3.5 (3.5)	4 (4)	4	5.2 (4.3)	4 (3.2)	7 (6)	3.9	4.7 (4)	5.4	4	4.8	4.7 (3)	4.3 (3.7)	4.5	4.2 (4.6)	5.2	5.5	6.8	5.4	6.8	4.5	4.8 (4)
	*H*	3.5 (3.5)	4.7 (4)	4.9	5.8 (4.5)	4.6 (3)	7 (6.2)	4.1	5.4 (4)	5.1	4	4.8	5.0 (4)	4.5 (3.1)	4.7	4.7 (4.2)	5.3	5.7	6.8	4.9	6.5	5.5	4.7 (4)
	*S*	3.8 (3.5)	3.8 (4)	7.2	8 (6.8)	4.3 (3)	7.3 (6.2)	7	5.5 (4)	7.4	4	7	6.4 (5.6)	4.8 (5.9)	7	4.8 (4.6)	7	6.4	6.8	4.7	6	6.6	4.8 (4)
	*P* value	ns^†^ (ns)	ns (ns)	***	*** (***)	ns (ns)	ns (ns)	***	ns (ns)	***	ns	***	* (***)	ns (***)^a^	***	ns (ns)	*	ns^b^	ns	ns	ns	*	ns (ns)

*Note*. Markers homozygous for the allele from the resistant parent PI 519805 are indicated with *R*, markers homozygous for the allele from the susceptible parent Avocet ‘S’ with *S*, and heterozygous markers with *H*. Underlined cells mark the recombination events. The *Yr78* phenotype was considered heterozygous when the segregating markers showed significant differences for the infection type (IT) scores and homozygous when the differences were not significant (statistical tests at the bottom of the table). Family size (*N*), average IT associated to each allele, and ANOVA *P* value are presented below the genotypes of each recombinant family. Results from the additional progeny tests performed in 2020–2021 are in parenthesis.

^a^ Retested in 2020–2021 and highly significant (*P* < .001, *n* = 32).

^b^ Although the *t* test was marginally not significant (*P* = .057), the mean values are consistent with the *H* phenotype suggested by all other recombinant lines.

*Significant at the .05 probability level.

***Significant at the .001 probability level.

†ns, nonsignificant.

In 2018, we genotyped and phenotyped all 1,721 plants, but in 2019 we genotyped 39 of the 54 F_3:4_ families (634 plants) and excluded 15 families that carried recombination events that were no longer informative based on the mapping results from 2018. The IT and SEV values were highly correlated (*R* = 0.954) (Supplemental Table S2).

For each of the genotyped families, we performed a one‐way ANOVA for the IT and SEV values using the classes defined by the segregating marker. We identified 86 families that showed significant differences in IT and SEV (*P* < .05) among the individuals carrying the segregating alleles (heterozygous), 37 families in which all the individuals were resistant and 36 families where all the individuals were susceptible. This segregation was not significantly different from a 1:2:1 segregation expected for a single resistance gene segregation (χ^2^ *P* = .76). The segregation of a single resistance gene in this population was also supported by the bimodal distribution of IT and SEV scores for both years (Supplemental Figure S1).

Using the more extensive genotypic and phenotypic data from 2018, we calculated the degree of dominance for *Yr78*. Plants heterozygous for the candidate gene region showed average IT (5.3) and SEV (53.1) values that were closer to the averages of the homozygous resistant plants (IT = 4.7, SEV = 47.7) than to the averages of the homozygous susceptible plants (IT = 7.6, SEV = 78.8). Using these values and Falconer's formula (Falconer, 1964), we estimated the degree of dominance of *Yr78* to be 0.62 for IT and 0.65 for SEV. The partial dominance of the resistant allele can be visualized in Figure 1c, which shows the frequency distribution of IT and SEV scores of the plants homozygous for the resistant allele (*R*), homozygous for the susceptible allele (*S*), or heterozygous (*H*) for the candidate gene region in the 1,721 plants genotyped and phenotyped in 2018.

### 3.2 High‐density map of *Yr78*

To dissect the 2.6‐cM candidate gene region between IWA7257 and IWA4408, we developed additional molecular markers. We first performed an exome capture to identify SNPs between the two parental lines PI 519805 and Avocet ‘S’. We obtained 100,816,796 150‐bp paired‐end reads for Avocet ‘S’ and 117,964,332 for PI 519805, which included 21% duplicates (Supplemental Table S3). We mapped 96% of the reads to the CS RefSeq v1.1 and identified a total of 6,323,811 unfiltered variants using the GATK pipeline.

On the basis of these SNPs, we developed 39 KASP markers equally spaced over the 27.52‐Mb region between IWA7257 and IWA4408. The primers used for these markers are described in Supplemental Table S4. We used these markers to genotype the 159 lines showing recombination events within the candidate region and to map *Yr78* more precisely within this interval. Several families with critical recombination events were planted again in 2020–2021, providing additional support to the proposed location of *Yr78*.

The genotypic data and the IT segregation for the critical recombination events closest to *Yr78* are presented in Table 1. Twenty of the 22 progeny tests showed statistical results consistent with the mapping position of *Yr78* between molecular markers CDM88 and CDM103, which are 11.16 Mb apart in the CS RefSeq v1.1. Although statistical tests for R130 and R148 were not significant in the first progeny test, their means were consistent with a family segregating for *Yr78* (lowest values for the homozygous resistant progeny, highest values for the homozygous susceptible progeny, and intermediate values for the heterozygous progeny). For R130, we performed a larger progeny test in 2020–2021 and detected highly significant differences among the genotypic classes (*P* < .001, *n* = 32). This result confirmed that this family is heterozygous for *Yr78* and consistent with the other progeny tests.

For family R148, we do not have data for a second experiment, but the *t* test between three plants homozygous for the susceptible allele (average IT = 6.4 ± 0.3) and the eight plants homozygous for the resistant allele (average IT = 5.5 ± 0.2) was only marginally nonsignificant (*P* = .057), suggesting insufficient statistical power rather than an inconsistent genotype. On the basis of the marginal *P* value, the consistency of the IT means with a family segregating for *Yr78*, and the independent information provided by the other 11 progeny tests with recombination events in the distal region (Table 1), we decided to classify R148 as heterozygous for *Yr78*. In summary, we mapped *Yr78* 0.02 cM proximal to CDM88 (on the basis of one recombination event in R162) and 0.03 cM distal to CDM103 (on the basis of two recombination events in R49 and R142).

### 3.3 Reduced recombination in the *Yr78* candidate regions and nucleolar organizing region mapping

**FIGURE 2:**
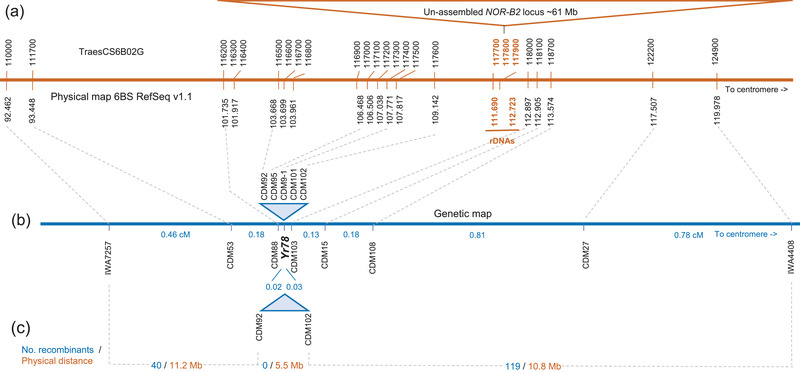
Relationship between physical and genetic distances in the IWA7257–IWA4408 interval. (a) Physical map of chromosome arm 6BS based on CS RefSeq v1.1. Coordinates in megabase (Mb) are indicated below the map and gene names on top (without the prefix TraesCS6B02G). The gene names in orange are misannotated pieces of ribosomal genes (Supplemental Table S5), and the orange triangle represents the unassembled *NOR‐B2* locus based on Handa et al. (2018). (b) Genetic map based on 6,124 segregating chromosomes. Primers for CDM markers are described in Supplemental Table S4. The blue triangle represents the region completely linked to *Yr78*. (c) Relationship between physical and genetic distances in the *Yr78* candidate gene region and its flanking regions

In the 5.5 Mb between markers CDM92 and CDM102, we did not find a single recombination event. By contrast, in the adjacent 11.2‐Mb distal interval between CDM92 and IWA7257, we detected 40 recombination events resulting in a ratio between genetic and physical distances of 3.6 recombination events per megabase (17.16 Mb cM^−1^). In the adjacent 10.8‐Mb proximal interval between CDM102 and IWA4408, we detected 119 recombination events resulting in a ratio of 11 recombination events per megabase (5.58 Mb cM^−1^) (Figure 2).

Since previous studies have shown reduced recombination around the nucleolar organizing regions (NORs) in wheat (Luo et al., 1998), we explored the location of the *NOR‐B2* locus relative to the *Yr78* candidate region. The 6BS chromosome arm is ∼320 Mb, so the *Yr78* candidate region is located at ∼30% of the arm length from the telomere, a position similar to that of the *NOR‐B2* locus in wheat cytogenetic maps (Badaeva et al., 2007). A more recent genomic study has placed the *NOR‐B2* locus on chromosome arm 6BS between 111.9 and 112.5 Mb (Handa et al., 2018), which is within the 11.16‐Mb candidate gene region between *Yr78* flanking markers CDM88 (101.735 Mb) and CDM103 (112.898 Mb) (Figure 2).

A BLASTN search of RefSeq v1.1 using a complete 18S‐5.8S‐28S unit (5,785 bp) (Supplemental Figure S2) revealed five complete and multiple truncated ribosomal RNA copies (at least 13 fragments between 700 and 4200 bp) on chromosome arm 6BS between 111.7 and 112.7 Mb, although one isolated 4,062‐bp fragment was found at 123.7 Mb (Supplemental Table S5). It has been estimated that the *NOR‐B2* locus has ∼6,800 ribosomal gene copies that span ∼61.2 Mb (Handa et al., 2018), suggesting that ∼60 Mb are not yet assembled in this region in RefSeq v1.1.

The assembled *NOR‐B2* region in RefSeq v1.1 includes three annotated genes, *TraesCS6B02G117700*, *TraesCS6B02G117800*, and *TraesCS6B02G117900*, which are incorrect translations of partial sequences of the 28S ribosomal subunit (Supplemental Table S5; Figure 2). A more recent assembly of the CS genome (v4) (Alonge et al., 2020) expanded the region between *TraesCS6B02G117800* and *TraesCS6B02G117900* by 1–2 Mb including 87 additional annotated genes. However, these genes encode proteins similar to *TraesCS6B02G117900* and, therefore, are likely incorrectly translated 28S ribosomal genes. In summary, our results indicate that the *NOR‐B2* locus is within the candidate gene region for *Yr78* , between 111.7 and 112.7 Mb in RefSeq v1.1. This position agrees with two previously published studies (Alonge et al., 2020; Handa et al., 2018).

### 3.4 Haplotype analysis

**FIGURE 3:**
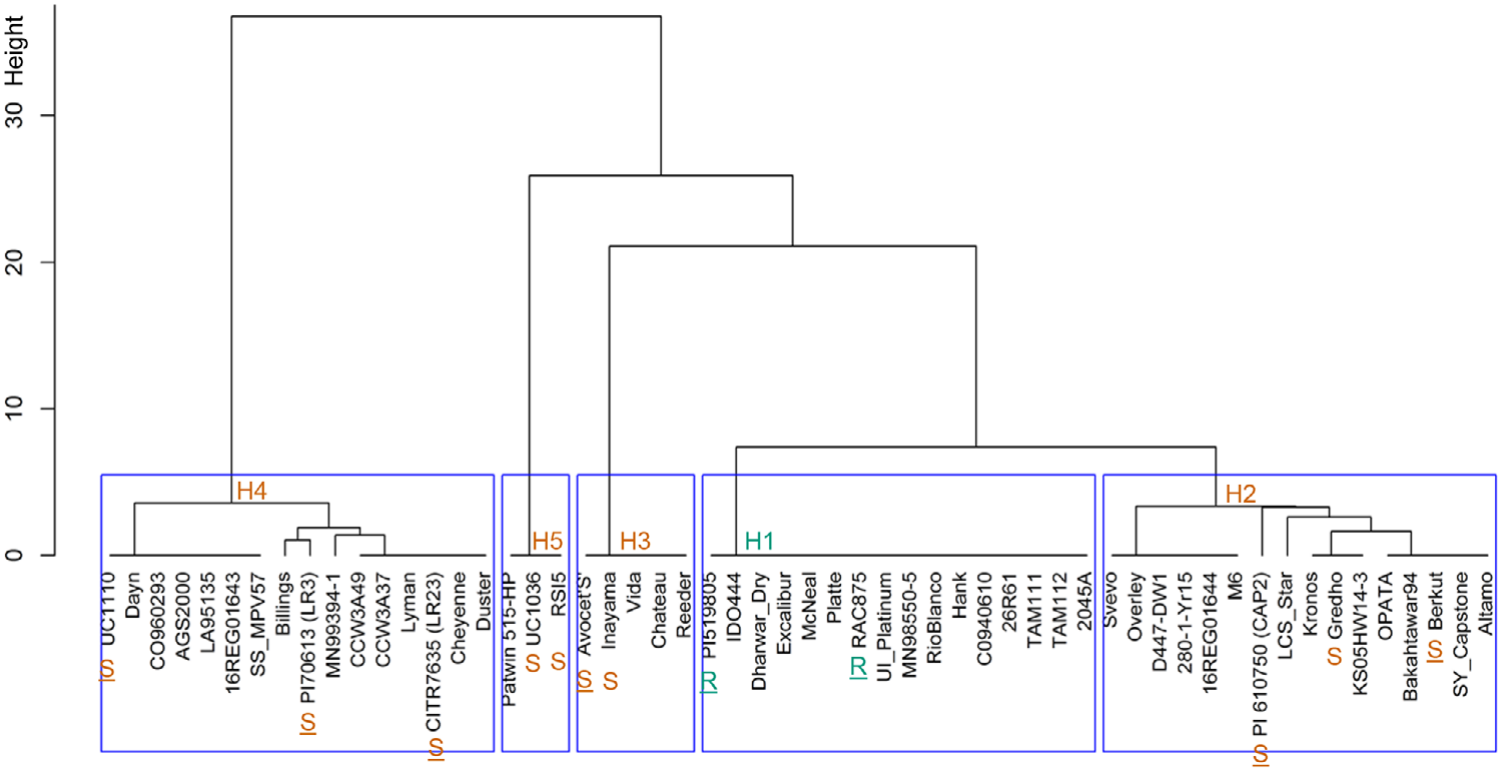
Haplotype analysis. Cluster analysis based on 204 single‐nucleotide polymorphisms reported in Supplemental Table S6. Haplotype 1 (H1) includes the two parental lines carrying the resistant allele of *Yr78* (R in green), whereas Haplotypes 2 and 3 (H2 and H3) include the parental lines of our mapping populations carrying the susceptible *Yr78* allele (S in orange). The underline below the S or the R indicates that the allele was confirmed by genetic data. Accessions tested as fully susceptible (>96% SEV) in California but without genetic data are indicated by an S without underline

We compared PI 519805 and Avocet ‘S’ exome capture data in the *Yr78* candidate region with similar data from other 53 tetraploid and hexaploid wheat cultivars extracted from the T3/Wheat database (see Material and Methods). Using the 204 SNPs identified in this region and a cluster analysis, we delimited five major clusters designated here as haplotypes H1 to H5 (Figure 3; Supplemental Table S6). Our *Yr78* resistant parental line PI 519805 belongs to haplotype H1, whereas the susceptible parental line Avocet ‘S’ was included in haplotype H3. This last haplotype includes the cultivar ‘Inayama’, which was previously shown to be very susceptible to stripe rust in field trials at Washington State University (2012–2014, IT = 9) and UC Davis (2012, IT = 7, SEV 90%). The Avocet ‘S’ and Inayama results confirmed that the H3 haplotype is associated with a susceptible allele of *Yr78*.

The H1 and H2 haplotypes are close to each other, so it was particularly important to determine if the H2 haplotype was associated with resistant or susceptible alleles of *Yr78*. The H2 tetraploid cultivar ‘Gredho’ (PI 532239) was tested during five seasons (2014–2018) at UC Davis, and it always showed SEV = 90–100% on the flag leaf. Similar results were reported in Pullman and Mt. Vernon in Washington state (IT = 8 and SEV = 99, https://npgsweb.ars‐grin.gov/gringlobal/accessiondetail?id=1427175), suggesting that the H2 haplotype in Gredho is associated with a susceptible allele of *Yr78*. We confirmed this hypothesis in a segregating F_5_ recombinant inbred line population from the cross Berkut (H2) × RAC875 (H1) genotyped with the 90K SNP Illumina assay and evaluated in 2016 for stripe rust at UC Davis (Supplemental Table S7). Lines carrying the Berkut allele (average IT = 7.4 ± 0.1 and SEV = 79.7 ± 2.3) were significantly more susceptible (*P <*.0001) than lines carrying the RAC875 allele (average IT = 4.5 ± 0.3 and SEV = 36.6 ± 4.2) (Supplemental Table S7). These results confirmed that the H2 haplotype is associated with a susceptible allele of *Yr78* and the H1 haplotype with the resistant allele.

A previously published stripe rust QTL study performed at UC Davis (Lowe et al., 2011) provided indirect evidence that the H4 haplotype is also associated with a susceptible allele of *Yr78*. In that study, four QTL for stripe rust resistance were identified on chromosomes 3B, 5A, 2B, and 2A in the cross UC1110 (H4) × PI 610750 (H2) with no evidence of resistance on chromosome 6B (Lowe et al., 2011). Plants without any of these QTL showed very high SEV (average 95%), suggesting that no additional resistance gene was present in this population. We sequenced the exome of a recombinant line from this population carrying the UC1110 allele at the *Yr78* candidate gene region and added this data to Supplemental Table S6. Analysis of this data showed that UC1110 has the H4 haplotype, suggesting that both the H2 and H4 haplotypes are associated with susceptible alleles for *Yr78*. This hypothesis was also supported by the lack of significant differences in IT and SEV between markers encompassing the *Yr78* candidate gene region in the crosses Berkut (H2) × CITR 7635 (H4) and Berkut (H2) × PI 70613 (H4) and by the high susceptibility of the parental lines CITR 7635 and PI 70613 in the field (IT = 7). Genotypic data from the 90K SNP Illumina assay (Blake et al., 2019) and stripe rust IT and SEV values obtained in a 2016 field experiment at UC Davis for these two populations are presented in Supplemental Tables S8 and S9.

We do not have mapping populations carrying haplotype H5, but the high susceptibility to stripe rust races (>96% SEV) reported in multiple regional trials in California for H5 cultivars RSI5 in 2001 (https://smallgrains.ucdavis.edu/2001/oct2001.htm) and UC1036 (later released as ‘Kern’) in 2003 (https://smallgrains.ucdavis.edu/2003/oct2003_apr286.htm) suggest that the H5 haplotype is also associated with a susceptible allele for *Yr78*.

### 3.5 Candidate genes in RefSeq v1.1

#### 3.5.1 Exome capture results

The *Yr78* candidate region between markers CDM88 and CDM103 includes 15 high‐confidence genes excluding the incorrectly annotated *TraesCS6B02G117700*, *TraesCS6B02G117800*, and *TraesCS6B02G117900* (fragments of 28S ribosomal RNA) and *TraesCS6B02G117500* (similar to a Harbinger DNA transposon) and including border genes *TraesCS6B02G116200* and *TraesCS6B02G118000* (Figure 2; Table 2). The coding regions of these two border genes are outside the candidate gene region, but the promoter of *TraesCS6B02G116200* and the 3′ untranslated and downstream region of *TraesCS6B02G118000* are within the candidate gene region and include open chromatin peaks detected by ATAC‐seq (assay for transposase‐accessible chromatin sequencing) from leaves (Lu et al., 2020) (Supplemental Figure S3). Since these open chromatin regions can include regulatory elements, we included these two genes in the list of candidate genes in Table 2.

**TABLE 2:** Predicted gene annotation in *Yr78* candidate region on wheat 6BS chromosome

Gene ID	Start	End	Strand	Gene ontology term	Annotation and predicted function
	bp				
*TraesCS6B02G116200* ^a^	101,731,455	101,735,467	−	GO:0009507	DUF1995. In *Arabidopsis*, this domain can be found in chloroplast protein LOW PSII ACCUMULATION 3 and in some putative adenylate kinases.
*TraesCS6B02G116300*	101,736,266	101,738,257	−	GO:0005515	PPRs (pentatricopeptide repeats) are sequence‐specific RNA‐binding proteins involved in multiple aspects of RNA metabolism such as RNA cleavage, splicing, stabilization, degradation, editing, and translation.
*TraesCS6B02G116400*	101,917,131	101,922,548	+	GO:0000272, GO:0016161	Beta‐amylase, producing maltose during hydrolytic starch degradation.
*TraesCS6B02G116500*	103,668,446	103,669,759	−	None	LRR (leucine‐rich repeat) domain, proteins containing LRRs are involved in signal transduction and immune response.
*TraesCS6B02G116600*	103,669,863	103,671,853	−	GO:0043531	RX (potato virus X resistance protein)‐like coiled‐coil, nucleotide‐binding site involved in plant resistance.
*TraesCS6B02G116700*	103,699,088	103,703,161	−	GO:0016616	L‐lactate dehydrogenase A pseudogene. ‘Chinese Spring’ has a 1‐bp deletion in first exon that is absent in other wheat sequenced genomes. When corrected, it restores a protein 100% identical to wild emmer XP_037452705.1.
*TraesCS6B02G116800*	103,960,679	103,965,406	−	GO:0016021	Reticulon‐like protein B23. May be involved in promoting membrane curvature or serving as endomplasmic reticulum‐associated channel‐like complexes.
*TraesCS6B02G116900*	106,467,784	106,469,793	+	GO:0005515	SWIB/MDM2 domain‐containing protein. Similar to SWI/SNF complex component SNF12 involved in transcriptional activation and repression of select genes by chromatin remodeling.
*TraesCS6B02G117000*	106,473,079	106,475,702	−	GO:0016788	Alpha/beta hydrolase superfamily protein. Has diverse biochemical activities in plant metabolism, hormone regulation and signaling, and defense.
*TraesCS6B02G117100*	106,506,094	106,512,307	+	GO:0006468, GO:0004672, GO:0005524	STK‐NBS‐LRR, containing a serine/threonine kinases domain, a nucleotide‐binding domain, and leucine‐rich repeat protein interaction domain. Usually associated with disease resistance.
*TraesCS6B02G117200*	107,038,087	107,038,635	−	GO:0008270	BLASTP shows no significant similarities outside wheat, and in wheat is repetitive (>50 matches, >85% identical over >90% length). Residues 22–62 similar to Znf_GRF domain involved in nucleic acid binding.
*TraesCS6B02G117300*	107,077,845	107,078,171	+	GO:0098869	*RESPONSE TO LOW SULFUR 3*, may be involved in plant responses to abiotic and biotic stress, prevents chloroplastic reactive oxygen species (ROS) production.
*TraesCS6B02G117400*	107,771,278	107,773,937	−	GO:0009638, GO:0016567	*NPH3* (*non‐phototropic hypocotyl 3*)/*RPT2* (*Root Phototropism 2*)‐like, assembles with CUL3 to form a E3 complex that ubiquitinates *Phototropin 1* (*phot1*) and modulates phototropic responsiveness.
*TraesCS6B02G117600* ^b^	109,141,595	109,143,509	+	GO:0042819, GO:0004359	Pyridoxal 5′‐phosphate synthase subunit PdxT/SNO, involved in vitamin B6 biosynthetic process.
*TraesCS6B02G118000* ^a^	112,897,909	112,903,050	−	GO:0004650, GO:0071555	Pectate lyase‐like superfamily protein, involved in cell wall organization.

^a^ The coding regions of these genes are outside the *Yr78* candidate gene region, but their potential regulatory regions are included.

^b^ *TraesCS6B02G117500* (DNA transposon) and *TraesCS6B02G117700*, *TraesCS6B02G117800*, and *TraesCS6B02G117900* (part of 28S ribosomal genes) were excluded.

The analysis of the conserved domains and predicted functions of the candidate genes (Table 2) revealed three genes with structures and functions typically associated with disease resistance. The first two, *TraesCS6B02G116600* (coiled coil [CC]‐NBS domain) and *TraesCS6B02G116500* (LRR domain), are annotated in RefSeq v1.1 as two separate genes separated by a 103‐bp intergenic region (− strand) because of the presence of a premature stop codon and a 2‐bp frame shift deletion. However, comparisons with other sequenced wheat genomes revealed that these two mutations are absent in the ‘Arina*LrFor*’, ‘CDC Stanley’, and ‘CDC Landmark’ genomes carrying the H4 haplotype (Supplemental Figure S4, Supplemental Table S10). In these cultivars, translation can continue through the short ‘intergenic region’ into the second gene encoding a complete CC‐NBS‐LRR protein with 941 amino acids, which is 83.6% similar to Tausch's goat grass resistant protein RGA5 (XP_020162986). Unfortunately, the incorrect annotation as two separate genes was transferred from CS to CDC Stanley, Arina*LrFor*, and CDC Landmark genomes despite the absence of the premature stop codon or the frame shift deletion, and this needs to be corrected in future annotations (Supplemental Figure S4). The G469* premature stop codon and the 2‐bp deletion are also absent in the three accessions with the H5 haplotype (Supplemental Table S6).

Using Sanger sequencing and exome capture, we confirmed that *TraesCS6B02G116600* and *TraesCS6B02G116500* are identical in the resistant parent PI 519805 (H1) and CS (H2). Both haplotypes have a premature stop codon at G469* (wrongly annotated as the end of *TraesCS6B02G116600*) and a 2‐bp deletion (CTT to C) that eliminates a phenylalanine at position 519 of the complete protein. To avoid this frame shift, which generates an additional premature stop codon, *TraesCS6B02G116500* was annotated as a separate gene. These mutations are present in all the sequenced genomes with H1 or H2 haplotypes (Supplemental Table S10). Avocet ‘S’ (H3) does not have G469* but has the 2‐bp deletion and a different premature stop codon (Supplemental Figure S4). These results indicate that this CC‐NBS‐LRR gene is truncated in both the resistant (H1) and susceptible (H2 and H3) parental lines of our mapping populations and that this gene is an unlikely candidate for *Yr78*.

The third gene with an architecture associated with disease resistance is *TraesCS6B02G117100*, which encodes a serine–threonine protein kinase (S–TPK)‐NBS‐LRR protein. The exome capture data revealed four SNPs, three of which resulted in amino acid changes (Supplemental Table S6). However, none of these SNPs differentiated the resistant accessions ‘RAC875’ and PI 519805 (H1) from the susceptible accessions Berkut (H2) and Avocet ‘S’ (H3) (Supplemental Table S6), suggesting that none of them were causal polymorphisms for the *Yr78* resistance.

Among the rest of the candidate genes, we detected 30 amino acid changes in the exome capture data (Supplemental Table S6), but only one of them differentiated the resistant H1 haplotype from most of the susceptible H2 haplotypes (except for PI 610750) (Supplemental Table S6). The SNP at 101,737,722 bp in gene *TraesCS6B02G116300* results in an amino acid change (R179M) predicted to have a moderate effect on protein structure and function. *TraesCS6B02G116300* encodes a protein with pentatricopeptide repeats, which are sequence‐specific RNA‐binding proteins involved in multiple aspects of RNA metabolism (Table 2).

#### 3.5.2 Genome comparison H1 vs. H2 haplotypes

The exome capture data can miss polymorphisms in regulatory regions and in genes not covered by the assay. Therefore, we used the wheat genomes sequenced in the PanGenome project (Walkowiak et al., 2020) to test for the presence of additional polymorphisms in the candidate genes and their putative regulatory regions. First, we used haplotype‐specific SNPs identified in Supplemental Table S6 to determine the haplotypes of the sequenced genomes in the candidate gene region. We determined that ‘Lancer’, ‘Cadenza’, and spelt [*T. aestivum* L. subsp. *spelta* (L.) Thell.] (PI 190962) have the H1 haplotype; CS, ‘Norin61’, ‘Mace’, ‘SY‐Mattis’, and ‘Jagger’ the H2 haplotype; and CDC Stanley, Arina*LrFor*, and CDC Landmark the H4 haplotype (Supplemental Table S10). Since our mapping results indicated that the H1 haplotype is associated with the *Yr78* resistant allele and the close H2 haplotype with a susceptible allele, we compared the candidate genes in the genomes of Lancer and Cadenza (H1) with those in CS and Norin61 (H2).

We first used this indirect strategy to explore the introns and regulatory region of the S–TPK‐NBS‐LRR gene *TraesCS6B02G117100*. We compared the complete gene including 6,214 bp in the introns, 1,038 bp in the promoter region, and 2,000 bp in the 3′ region downstream of the stop codon. We did not expand the promoter analysis beyond 1,038 bp because of the presence of large retroelements beyond this point (Supplemental Table S11). All these regions were identical in the genomes with the H1 and H2 haplotypes, indicating that they are unlikely to contribute to the differences in resistance observed between these haplotypes. However, we cannot rule out the presence of polymorphisms in distant regulatory elements not included in the explored region.

We also used this strategy to explore the regulatory regions of the two border genes, which have their coding sequences outside of the candidate gene region but their regulatory regions inside. The 798 bp in the *TraesCS6B02G116200* promoter region (before reaching the end of *TraesCS6B02G116300*) were identical in the H1 and H2 genomes. Similarly, the 2 kb downstream of the stop codon in *TraesCS6B02G118000* were almost identical among the H1 and H2 haplotypes except for one A/G SNP at position 112,895,915. Chinese Spring and other H2 genomes have the A allele, whereas the genomes with H1 and H4 haplotypes carried the G allele (Supplemental Table S11). Since H4 is a susceptible haplotype, this SNP is very unlikely to be responsible for the differences in resistance associated with *Yr78*. In summary, the comparison of these two regulatory regions failed to reveal major polymorphisms between H1 and H2 suggestive of changes in gene expression in these two border genes.

Finally, we compared the coding regions, introns, and 2 kb upstream and downstream of all the candidate genes between the H1 and H2 genomes. In the coding regions, we detected a 1 bp deletion (between 103,703,065 – 103,703,064) in the first exon of *TraesCS6B02G116700*. This frame‐shift mutation, which is present only in CS among the genomes with the H2 haplotype (Supplemental Table S10), likely caused the incorrect annotation of this gene in CS RefSeq v1.1. Adding the missing C to the CS pseudogene results in the correct translated protein, which is identical to the L‐lactate dehydrogenase A present in the other haplotypes (XP_037452705).

The genomic comparison revealed the presence of a polymorphism (SNP 107,077,900) in *TraesCS6B02G117300*, which was not detected in the exome capture. This gene encodes a protein similar to RESPONSE TO LOW SULFUR 3, which has been shown to be a target of pathogen effectors and to affect resistance reactions to different pathogens (Garcia‐Molina et al., 2017). This polymorphism resulted in an amino acid change (G19D) that differentiated all the sequenced genomes with the H1 haplotype from those with the H2 and H4 haplotypes (Supplemental Table S10). Comparison of this gene across the different genomes revealed two additional nonsynonymous SNPs described in Supplemental Table S10. The rest of the candidate genes showed no polymorphisms between the genomes of the accessions carrying the H1 and H2 haplotypes (Supplemental Table S11).

The promoter and 3′ regions (∼24 kb analyzed) showed only four SNPs and one 9‐bp indel differentiating the genomes with the H1 haplotype from those carrying the H2 and H4 haplotypes. These changes were detected in *TraesCS6B02G116400*, *TraesCS6B02G116700*, *TraesCS6B02G117400*, and *TraesCS6B02G117600* and are described in Supplemental Table S11. In addition to their value as potential causal polymorphisms, these SNPs can potentially be used to develop additional diagnostic markers for the H1 haplotype. The paucity of SNPs between H1 and H2 confirmed the close evolutionary relationship between these two haplotypes and supported the cluster analysis presented in Figure 3 on the basis of the exome capture results.

In summary, the functional annotation of the candidate genes and the polymorphisms between resistant and susceptible haplotypes provided useful information to prioritize some of these genes for future functional characterization.

### 3.6 Diagnostic SNPs for resistant haplotype H1

Marker IWA7257 (RefSeq v1.1 92,462,100) was mapped in a previous study 0.6 cM distal to *Yr78* (Dong et al., 2017), a genetic distance confirmed in this study (0.66 cM) (Figure 2). The inclusion of IWA7257 in the haplotype analysis (Supplemental Table S6) showed that this marker differentiates well most of the accessions carrying the H1 and H2 haplotypes. However, lines 26R61 and C0940610 show a historic recombination event separating IWA7257 from the *Yr78* candidate gene region, suggesting that better diagnostic markers for *Yr78* can be generated from the region completely linked to this resistance gene.

For this purpose, we selected two SNPs that discriminated the H1 accessions from the H2, H3, H4, and H5 accessions (Supplemental Table S6) and developed codominant KASP markers CDM158 (SNP‐106,540,703) and CDM160‐2 (SNP‐108,227,904). We tested these markers in PI 519805, Avocet ‘S’, heterozygous plants from the progeny test, 10 diverse lines that were previously shown to carry the resistant *Yr78* allele (Dong et al., 2017) (Supplemental Table S12A) and 22 lines that were highly susceptible to the stripe rust races present in California (Maccaferri et al., 2015) (Supplemental Table S12B).

**FIGURE 4:**
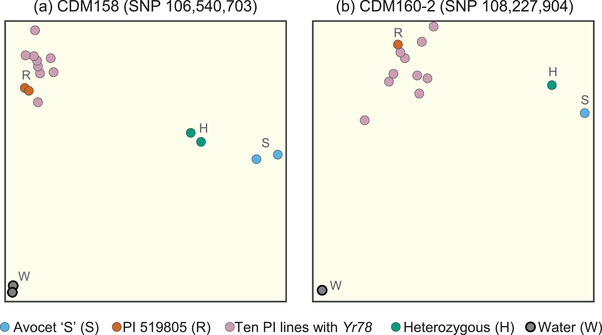
Diagnostic markers for the H1 haplotype carrying the resistant allele of *Yr78*. (a) CDM158 (single‐nucleotide polymorphism (SNP) 106,540,703) and (b) CDM160‐2 (SNP 108,227,904)

Both diagnostic markers showed a clear clustering of the 10 *Yr78* PI accessions (Figure 4, pink dots) with the resistant parental line PI 519805 (Figure 4, red dots), confirming the presence of *Yr78* in the 10 previously published lines. The susceptible allele from Avocet ‘S’ (Figure 4, blue dots) was well separated from the H1 cluster, whereas the heterozygous lines (Figure 4, green dots) showed an intermediate position. None of the highly susceptible lines had the alleles characteristic of the H1 haplotype for these markers (Supplemental Table S12B). These results indicate that the combined use of these two SNPs provides a confident prediction of the presence of the H1 haplotype.

## 4 DISCUSSION

### 4.1 Effect of the *NOR‐B2* locus on the map‐based cloning of *Yr78*

In this study, we mapped the stripe rust resistance gene *Yr78* within an 11.16‐Mb interval in CS RefSeq v1.1 including the *NOR‐B2* locus. Early studies in wheat showed that the number of ribosomal DNA (rDNA) units in the *NOR‐B2* locus is variable in wheat, ranging from 5,500 copies in CS (∼50 Mb) to 2,000 copies in the cultivar ‘Holdfast’ (Flavell & O'Dell, 1976). A more recent study estimated that the number of rDNA units in CS is closer to 6,800 copies, spanning a region of ∼61 Mb (Handa et al., 2018). Since the *NOR‐B2* locus is included in the *Yr78* candidate region, the haplotypes defined in this study provide a useful framework for future studies aimed at the characterization of the natural variation in the *NOR‐B2* locus in wheat.

Our analysis of rDNA units on CS chromosome 6B showed that they span only a 1‐Mb region in RefSeq v1.1 (Supplemental Table S5), suggesting that ∼60 Mb of rDNA subunits remain to be assembled in this region. Although most of the unassembled region is likely to be composed of rDNA units, we cannot completely rule out the presence of other genes, introducing some uncertainty on the completeness of the list of potential candidate genes for *Yr78* presented in this study. This result highlights the importance of closing these large gaps in the wheat reference genome.

The presence of the *NOR‐B2* locus within the *Yr78* candidate gene region introduces an additional complication because of its negative effect on recombination. Significantly reduced recombination rates have been observed within NOR loci in yeast (*Saccharomyces cerevisiae*) (Petes, 1979), *Drosophila melanogaster* (Williams & Robbins, 1992), and maize (*Zea mays* L.) (Simcox et al., 1995). In addition, the presence of the NOR loci in wheat have been associated with reduced recombination rates in the regions flanking these loci (Luo et al., 1998). A similar phenomenon was observed in our study, where we found only two recombination events in 6,124 segregating chromosomes between markers CDM102 (109.1 Mb) and CDM103 (112.9 Mb) flanking the *NOR‐B2* locus (Figure 2). Although we do not know the exact location of these two recombination events, this result indicates significantly reduced or no recombination within the *NOR‐B2* locus.

Ratios between genetic and physical distances differed between the distal and proximal regions flanking the *NOR‐B2* locus. In the distal region, we failed to find any recombination event in the 5.5 Mb between CDM92 and CDM102, whereas in the proximal region, we detected recombination between the first two genes located <0.2 Mb from the last rDNA cluster (Figure 2). This asymmetry in ratios between physical and genetic distances extended further. In the distal region (IWA7257, *NOR‐B2*), the ratio was 29.1 Mb cM^−1^, whereas in the proximal region (*NOR‐B2* , IWA4408) the ratio was 3.8 Mb cM^−1^. We currently do not know the reason for this difference. Since none of the currently available wheat genomes carry the H3 haplotype (Supplemental Table S10), we cannot rule out the possibility of unknown structural changes between the H1 and H3 haplotypes affecting the recombination rate in the distal region.

In summary, the presence of the *NOR‐B2* locus within the *Yr78* candidate gene region represents a significant problem for the cloning of this resistance gene using a map‐based cloning approach, both for the reduced recombination in the region and for the absence of a complete genome reference in the region.

### 4.2 Haplotype analysis and origin of *Yr78*

The haplotype analysis based on the exome capture data revealed five major groups with different degrees of relatedness and frequencies (Figure 3; Supplemental Table S6). The two haplotypes with the lowest frequency, H3 and H5, are not represented in the currently sequenced genomes (Supplemental Table S10). The H5 haplotype was found in three cultivars from California, representing 5% of the accessions included in the exome capture but was much lower (0.5%) in the published 1000‐exomes study (He et al., 2019) (Supplemental Table S13). This difference may be generated by an overrepresentation of H5 in California cultivars or by an underestimation of the H5 frequency in the 1000‐exomes study because of SNP filtering for minor allele frequency. Since two of these cultivars (RSI5 and UC1036/Kern) became highly susceptible to the post‐2000 stripe rust races, we assume that H5 does not carry a functional *Yr78* allele.

The H3 haplotype, which includes the susceptible parental line Avocet ‘S’, was found in 9% of the accessions in the exome capture and in 7% of the accessions in the 1000‐exomes project, suggesting a relatively low frequency (Supplemental Tables S13 and S14). The H4 haplotype includes line UC1110, which was shown to have no stripe rust resistance genes on chromosome 6B (Lowe et al., 2011) and is, therefore, unlikely to carry *Yr78*. The H4 haplotype was detected in 28.1% of the accessions in the exome capture and 30.5% of the accessions in the 1000‐exomes project (Supplemental Tables S13 and S14) and is also present in the sequenced genomes of CDC Stanley, CDC Landmark, and Arina*LrFor* (Supplemental Table S10).

Finally, the closely related haplotypes H1 and H2 were the most abundant haplotypes with frequencies between 28 and 32% in both the exome capture and 1000‐exomes (Supplemental Tables S14). Interestingly, in the latter study, the frequency of the H1 haplotype was higher among commercial cultivars (36.1%, *n* = 407) than among landraces (20.2%, *n* = 277), suggesting the possibility of positive selection for the resistant allele. This hypothesis is also supported by the absence of the H1 haplotype in 393 accessions of durum wheat tested with markers CDM158 and CDM160‐2 (Supplemental Table S15), suggesting a rapid increase in the frequency of the H1 haplotype in hexaploid wheat. We also failed to detect the H1 haplotype in 88 accessions of wild emmer and 92 accessions of emmer wheat (Supplemental Table S15), suggesting a possible origin of the H1 haplotype and *Yr78* in hexaploid wheat.

The presence of the H1 allele in the genome of spelt (PI 190962, a stripe rust resistant accession from Italy, https://npgsweb.ars‐grin.gov/gringlobal/accessiondetail?id=1162413) suggested that *Yr78* may have originated in European spelt wheat. To explore this possibility, we characterized the 12 spelt accessions included in the 1000‐exomes study and 49 additional accessions provided by Dr. Jan Dvorak (UC Davis) (Supplemental Table S14). For the accessions in the 1000‐exomes study, we were able to infer the exact haplotype. The other spelt accessions were genotyped with the two H1 diagnostic markers, so we were able to determine only if they carry the H1 haplotype or not. None of the nine spelt wheat from Asia had the H1 haplotype (the one found in the 1000‐exome showed the H4 haplotype). By contrast, 67.3% of the 52 European spelt had the H1 haplotype (the four non‐H1 accessions found in the 1000‐exome study showed the H2 haplotype) (Supplemental Table S14).

These results support the known polyphyletic origin of the European and Asian spelt wheats (Dvorak et al., 2012). Since the European spelt wheat likely originated from hybridization between free‐threshing hexaploid wheat and hulled cultivated emmer (Dvorak et al., 2012), we explored our collection of 92 cultivated emmer accessions. The H1 haplotype was not detected in these accessions, so we hypothesize that *Yr78* originated either in the European spelt wheat or was introgressed from its hexaploid progenitor. A larger screen of a cultivated emmer collection will be required to validate this hypothesis. The origin of *Yr78* is further complicated by the presence of one H1 accession in club wheat [*T. aestivum* L. subsp. *compactum* (Host) MacKey] and two H1 accessions in macha wheat [*T. aestivum* L. subsp. *macha* (Dekapr. & Menabde) MacKey] (Supplemental Table S14). Irrespective of its exact origin, the presence of *Yr78* in all four subspecies of hexaploid wheat points to an ancient origin of *Yr78*.

The high frequency of H2 in bread wheat, together with its presence in durum wheat, and the higher variability observed within H2 relative to other haplotypes (Supplemental Table S6) points to H2 as the ancestral haplotype. As examples of the internal variability in H2, CS and ‘LCS‐Star’ carry three SNPs that differentiate them from all other haplotypes (Supplemental Table S6), whereas a group of unique SNPs were shared by the H2 genomes of SY‐Mattis, ‘Julius’, ‘Claire’, and ‘Rubigus’ (Supplemental Table S10).

### 4.3 Candidate genes linked to *Yr78*

Although the absence of a complete assembly of the *NOR‐B2* locus introduces some uncertainties into the list of potential candidate genes, we decided to prioritize the annotated genes for future functional characterization studies. For this, we used two different criteria: the functional annotation of the candidate genes and the predicted effects of the polymorphism between the resistant and susceptible haplotypes.

#### 4.3.1 Candidate genes prioritized based on annotated function

The discovery of a CC‐NBS‐LRR gene within the *Yr78* candidate region was a promising result because this gene architecture is frequent in disease resistance genes. However, further analyses showed that both PI 519805 and the three sequenced genomes carrying the H1 haplotype have a premature stop codon (G469*) and a 2‐bp frame‐shift deletion that result in a protein lacking the 3′ half and that is most likely nonfunctional. Avocet ‘S’ does not have the G469* premature stop codon, but it still has the 2‐bp deletion that causes a secondary stop codon and a truncated and likely nonfunctional protein (Supplemental Figure S4). The two mutations were detected in all genomes carrying the H1 and H2 haplotypes but were both absent in the genomes carrying the H4 haplotype (and H5 exome capture), where *TraesCS6B02G117500‐600* encodes a single complete CC‐NBS‐LRR. These results indicate that this CC‐NBS‐LRR gene is truncated and likely not functional in both the resistant and susceptible parental lines in our mapping populations. On the basis of these results, we conclude that *TraesCS6B02G117500‐600* is an unlikely candidate gene for *Yr78*.

The other prioritized gene based on its annotation is *TraesCS6B02G117100*, which encodes a protein with N‐terminal S–TPK and C‐terminal NBS‐LRR domains. This gene architecture is frequent in wheat and has been associated with different disease resistance genes (Afzal et al., 2008; Andersen et al., 2020; Faris et al., 2010). Our exome capture results and the comparison of genomes carrying the H1 (resistant) and H2 (susceptible) haplotypes failed to reveal any SNPs in the coding region, introns, promoter, or 3′ region of *TraesCS6B02G117100* (Supplemental Table S6 and S11). However, since we cannot rule out the possibility of regulatory polymorphisms outside the investigated region but within the candidate gene region, we have not eliminated this gene from the list of prioritized genes for future expression studies and functional characterization.

#### 4.3.2 Candidate genes prioritized based on polymorphisms between resistant and susceptible haplotypes

Within the candidate gene region, we detected two nonsynonymous polymorphisms between the H1 and H2 haplotypes. The first one was an R179M polymorphism with a predicted moderate effect on the structure and function of the pentatricopeptide repeats protein encoded by *TraesCS6B02G116300*. This polymorphism was detected in all 19 accessions with the H1 haplotype, but it was also present in a few accessions carrying the H2 haplotype (PI 610750 in Supplemental Table S6 and ‘Paragon’ in Supplemental Table S10). We previously established that both PI 610750 and UC1110 carried susceptible *Yr78* alleles based on the highly susceptible reaction (SEV = 95%) observed in plants lacking any of the four QTL for stripe rust resistance identified on chromosomes 3B, 5A, 2B, and 2A in a cross between these two lines (Lowe et al., 2011). Therefore, it is highly unlikely that the R179M represents the causal polymorphism for *Yr78*.

The second nonsynonymous polymorphism between accessions carrying the H1 and H2 haplotypes was detected in the comparative genomics analysis in *TraesCS6B02G117300* (Supplemental Table S10 and S11). The G19D polymorphism in the protein RESPONSE TO LOW SULFUR 3‐like encoded by this gene was consistent across the genomes (Supplemental Table S10). The *Arabidopsis thaliana* (L.) Heynh. homologous proteins, LOW SULPHUR UPREGULATED (LSU), are targeted by virulence effectors from diverse pathogens and are upregulated in several abiotic and biotic stress conditions (Garcia‐Molina et al., 2017; Mukhtar et al., 2011; Wessling et al., 2014). The *LSU1* gene overexpression confers significant disease resistance under several abiotic stresses suggesting that it plays an important role in the coordination of plant immune responses and abiotic stress (Garcia‐Molina et al., 2017). On the basis of the consistent amino acid polymorphism between the H1 and H2 haplotypes and its potential role in disease resistance responses, we prioritized this gene for future functional characterization.

The other five SNPs between the H1 and H2 haplotypes were identified in the flanking 2‐kb regions in the promoter (three SNPs and one indel) or the 3′ region after the stop codon (two SNPs, Supplemental Table S11). Additional studies of the expression of these genes in infected and mock‐inoculated leaves from plants segregating for the H1 and H2 haplotypes (e.g., Berkut × RAC875 recombinant inbred lines) will be necessary to determine if these mutations have any effect on the regulation of these genes.

### 4.4 Implications of this study on the deployment of *Yr78* in wheat breeding programs

The stripe rust pathogen is established in more than 60 countries (Chen, 2020) and it continues to be an important threat to wheat production. In 2021, high stripe rust pressure was reported in China (Zhou et al., 2021) and in the United States, where the Cereal Rust Bulletin reported severe wheat stripe rust in most of the Plains states and western Washington (https://www.ars.usda.gov/midwest‐area/stpaul/cereal‐disease‐lab/docs/cereal‐rust‐bulletins/cereal‐rust‐bulletins/). The global cost of controlling this disease exceeds US$ 1 billion annually (Chen, 2020), providing a practical rationale for the search for genes conferring a broad resistance to this pathogen.

*Yr78* has remained effective in California under heavy *Pst* pressure since its discovery 10 years ago, and its effectiveness likely extends much longer. *Yr78* has been recently reported to be present in the widely grown Pacific Northwest (PNW) winter wheat cultivars ‘Madsen’ (PI 511673, released 1988) and ‘Stephens’ (CItr 17596, released in 1977), where it was originally identified as QTL *QYrMa.wgp‐6BS* and *QYr.wgp‐6BS*, respectively (Dong et al., 2017; Liu et al., 2018; Santra et al., 2008). These high‐temperature adult‐plant resistance QTL have protected wheat from stripe rust for >40 yr in the Pacific Northwest, which is likely sufficient to designate *Yr78* as a durable disease resistance gene. The presence of the H1 haplotype in more than half of the European spelt analyzed in this study suggests that this gene may have been protecting wheat from stripe rust for hundreds of years.

The precise mapping of *Yr78* and the identification of the tightly linked haplotype H1 allowed us to develop two diagnostic markers. Markers CDM158 and CDM160‐2 are useful tools for wheat breeders and researchers to establish the distribution of *Yr78* in their germplasm and plan informed crosses to accelerate the deployment of this resistance gene. These two markers represent an improvement over IWA7257 because historic recombination events were detected between this marker and *Yr78* (Supplemental Table S6), which reduces the predictive value of IWA7257. Given the limited recombination detected in the *Yr78* region, a single diagnostic marker would be sufficient to predict the presence of the resistance gene, but we developed a second marker as a precaution.

These linked markers will also be useful to introgress *Yr78* into tetraploid wheat. Our survey of 393 durum and 180 emmer accessions failed to detect the H1 haplotype, suggesting that *Yr78* has not been used before in pasta wheat breeding. Therefore, the introgression of *Yr78* into durum wheat represents a unique opportunity to increase pasta wheat resistance to stripe rust in most durum wheat germplasm. We have initiated crosses between Cadenza and tetraploid wheat Kronos to initiate this process.

In summary, this study revealed a close linkage between *Yr78* and the *NOR‐B2* locus and delimited a small region where candidate genes were prioritized for future functional characterization based on their known function in other plant species and on their polymorphisms between resistant and susceptible haplotypes. However, additional studies will be necessary to determine if additional candidate genes are present in the unassembled *NOR‐B2* region. Despite the pending tasks for the final identification of the causal gene, this study completed the characterization of the haplotype associated with *Yr78* and the development of two diagnostic markers that will accelerate the deployment of *Yr78* in both pasta and bread wheat breeding programs.

## AUTHOR CONTRIBUTIONS

Chen Dang: Formal analysis; Investigation; Methodology; Writing – original draft; Writing – review & editing. Junli Zhang: Formal analysis; Investigation; Methodology; Software; Writing – review & editing. Jorge Dubcovsky: Conceptualization; Data curation; Formal analysis; Funding acquisition; Methodology; Project administration; Resources; Supervision; Visualization; Writing – review & editing.

## CONFLICT OF INTEREST

The authors declare that they do not have any conflict of interest.

## Supporting information

Supplemental Figures
Supplemental Figure S1: Distribution of IT and SEV scores in 2018 and 2019.
Supplemental Figure S2: Sequence of ribosomal genes from chromosome arm 6BS used to blast the CS RefSeq v1.1 genome.
Supplemental Figure S3: Genes at the border of the *Yr78* candidate gene region.
Supplemental Figure S4: Sequence analyses supporting *TraesCS6B02G116500* and *TraesCS6B02G116600* (RefSeq v1.1) as a single pseudogene.

SUPPLEMENTAL MATERIALS
Supplemental Tables
Supplemental Table S1: Evaluation of parental lines Avocet ‘S’ and PI 519805 for stripe rust resistance.
Supplemental Table S2: Genotyping for *Yr78* flanking markers and phenotyping for stripe rust resistance at UC Davis in 2018.
Supplemental Table S3: Exome capture statistics.
Supplemental Table S4: KASP markers used for high density mapping of *Yr78*.
Supplemental Table S5: Ribosomal RNA genes or partial genes coordinates in CS 6BS RefSeq v1.1.
Supplemental Table S6: Haplotype analysis of the *Yr78* candidate gene region.
Supplemental Table S7: IT and severity reactions to stripe rust in a segregating population from the cross Berkut (H2) × RAC875 (H1).
Supplemental Table S8: IT and severity reactions to stripe rust in a segregating population from the cross Berkut (H2) × PI 70613 (H4).
Supplemental Table S9: IT and severity reactions to stripe rust in a segregating population from the cross Berkut (H2) × CITR 7635 (H4).
Supplemental Table S10: Nonsynonymous and differential haplotype SNPs identified in the wheat sequenced genomes.
Supplemental Table S11: Comparison of candidate genes between Lancer/Cadenza (H1) and CS/Norin61 (H2).
Supplemental Table S12: Validation of H1 diagnostic markers in sets of resistant and susceptible lines.
Supplemental Table S13: Haplotype analysis of the *Yr78* candidate region using 1000‐exome capture data.
Supplemental Table S14: Metadata for the 1000‐exome capture and frequencies of the *Yr78* haplotypes in cultivars and landraces.
Supplemental Table S15: Frequency of the H1 haplotype in three tetraploid wheat subspecies.

## Data Availability

All the exome capture data is available in T3/Wheat under the dataset WheatCAP_2017_UCD. All accessions used in this study are available from the USDA–ARS National Small Grains Collection. All the sequence information and the primers used in this study are available in the Supplemental Tables.
