# Supplementary material for: High‐resolution mapping of Yr78, an adult plant resistance gene to wheat stripe rust
Source: Plant Genome. 2022 Apr 25;15(2):e20212. doi: 10.1002/tpg2.20212 (PMC12806896; doi:10.1002/tpg2.20212)
Supplement: Supplementary file 1 [file TPG2-15-e20212-s002.docx]

**Supplemental Figures**

**Supplemental Figure S1.** **Distribution of IT and SEV scores in 2018 and 2019.**

**Supplemental Figure S2.** **Sequence of ribosomal genes from chromosome arm 6BS used to blast the CS RefSeq v1.1 genome.** Green **=** 18S, purple = 5.8S and blue = 28S.

TAGTCATATGCTTGTCTCAAAGATTAAGCCATGCATGTGCAAGTATGAACCAATTTGAACTGTGAAACTGCGAATGGCTCATTAAATCAGTTATAGTTTGTTTGATGGTACGTGCTACTCGGATAACCGTAGTAATTCTAGAGCTAATACGTGCAACAAACCCCGACTTCTGGGAGGGGCGCATTTATTAGATAAAAGGCTGACGCGGGCTCTGCTCGCTGATCCGATGATTCATGATAACTCGACGGATCGCACGGCCTTCGTGCCGGCGACGCATCATTCAAATTTCTGCCCTATCAACTTTCGATGGTAGGATAGGGGCCTACCATGGTGGTGACGGGTGACGGAGAATTAGGGTTCGATTCCGGAGAGGGAGCCTGAGAAACGGCTACCACATCCAAGGAAGGCAGCAGGCGCGCAAATTACCCAATCCTGACACGGGGAGGTAGTGACAATAAATAACAATACCGGGCGCATTAGTGTCTGGTAATTGGAATGAGTACAATCTAAATCCCTTAACGAGGATCCATTGGAGGGCAAGTCTGGTGCCAGCAGCCGCGGTAATTCCAGCTCCAATAGCGTATATTTAAGTTGTTGCAGTTAAAAAGCTCGTAGTTGGACCTTGGGCCGGGTCGGCCGGTCCGCCTCACGGCGAGCACCGACCTACTCGACCCTTCGGCCGGCATCGCGCTCCTAGCCTTAATTGGCCGGGTCGTGTTTCCGGCATCGTTACTTTGAAGAAATTAGAGTGCTCAAAGCAAGCCATCGCTCTGGATACATTAGCATGGGATAACATCATAGGATTCCGGTCCTATTGTGTTGGCCTTCGGGATCGGAGTAATGATTAATAGGGACAGTCGGGGGCATTCGTATTTCATAGTCAGAGGTGAAATTCTTGGATTTATGAAAGACGAACAACTGCGAAAGCATTTGCCAAGGATGTTTTCATTAATCAAGAACGAAAGTTGGGGGCTCGAAGACGATCAGATACCGTCCTAGTCTCAACCATAAACGATGCCGACCAGGGATCGGCGGATGTTGCTTATAGGACTCCGCCGGCACCTTATGAGAAATCAAAGTCTTTGGGTTCCGGGGGGAGTATGGTCGCAAGGCTGAAACTTAAAGGAATTGACGGAAGGGCACCACCAGGCGTGGAGCCTGCGGCTTAATTTGACTCAACACGGGGAAACTTACCAGGTCCAGACATAGCAAGGATTGACAGACTGAGAGCTCTTTCTTGATTCTATGGGTGGTGGTGCATGGCCGTTCTTAGTTGGTGGAGCGATTTGTCTGGTTAATTCCGTTAACGAACGAGACCTCAGCCTGCTAACTAGCTATGCGGAGCCATCCCTCCGCAGCTAGCTTCTTAGAGGGACTATCGCCGTTTAGGCGACGGAAGTTTGAGGCAATAACAGGTCTGTGATGCCCTTAGATGTTCTGGGCCGCACGCGCGCTACACTGATGTATTCAACGAGTATATAGCCTTGGCCGACAGGCCCGGGTAATCTTGGGAAATTTCATCGTGATGGGGATAGATCATTGCAATTGTTGGTCTTCAACGAGGAATGCCTAGTAAGCGCGAGTCATCAGCTCGCGTTGACTACGTCCCTGCCCTTTGTACACACCGCCCGTCGCTCCTACCGATTGAATGGTCCGGTGAAGTGTTCGGATCGCGGCGACGGGGGCGGTTCGCCGCCCCCGACGTCGCGAGAAGTCCATTGAACCTTATCATTTAGAGGAAGGAGAAGTCGTAACAAGGTTTCCGTAGGTGAACCTGCGGAAGGATCATTGTCGTGACCCTGACCAAAACAGACCGCGCACGCGTCATCCAATCCGTCGGCGACGGCACCGTCCGTCGCTCGGCCAATGCCTCGACCACCTCCCCTCCTCGGAGCGGGTGGGGGCTCGGGGTAAAAGAACCCACGGCGCCGAAGGCGTCAAGGAACACTGTGCCTAACCCGGGGGCATGTCTAGCTTGCTAGCCGTCCCTCGTGTTGCAAAGCTATTTAATCCACACGACTCTCGGCAACGGATATCTCGGCTCTCGCATCGATGAAGAACGTAGCGAAATGCGATACCTGGTGTGAATTGCAGAATCCCGCGAACCATCGAGTCTTTGAACGCAAGTTGCGCCCGAGGCCACTCGGCCGAGGGCACGCCTGCCTGGGCGTCACGCCAAAACACGCTCCCAACCACCCTCAACGGGATTCGGGATGCGGCATCTGGTCCCTCGTCTCTCAAGGGACGGTGGACCGAAGATTGGGCTGCCGGCGTACCGCGCCGGACACAGCGCATGGTGGGCGTCCTCGCTTTATCAATGCAGTGCATCCGGCGCGCAGCTGGCATTATGGCCTTTGAACGACCCAACAAACGAAGCGCACGTCGCTTCGACCGCGACCCCAGGTCAGGCGGGACTACCCGCTGAGTTTAAGCATATAAATAAGCGGAGGAGAAGAAACTTACAAGGATTCCCCTAGTAACGGCGAGCGAACCGGGAGCAGCCCAGCTTGAGAATCGGGCGGCTGTGCCGTCCGAATTGTAGTCTGGAGAGGCGTCCTCAGCGACGGACCGGGCCCAAGTCCCCTGGAAAGGGGCGCCTGGGAGGGTGAGAGCCCCGTCCGGCCCGGACCCTGTCGCCCCACGAGGCGCCGTCAACGAGTCGGGTTGTTTGGGAATGCAGCCCAAATCGGGCGGTAGACTCCGTCCAAGGCTAAATACATGCGAGAGACCGATAGCGAACAAGTACCGCGAGGGAAAGATGAAAAGGACTTTGAAAAGAGAGTCAAAGAGTGCTTGAAATTGCCGGGAGGGAAGCGGATGGGGGCCGGCGATGCGCCCCGGCCGTATGCGGAACGGCTCTTGCTGGTCCGCCGCTCGGCTCGGGGTGTGGACTGTTGTCGGCCGCGCCGGCGGCCAAAGCCCGGGGGCCCTAGGTGCCCCCGGTGGCCGTCGTCGGCACGGCCGGTACCCGCGCGCCTAAAGGCGTGTCCCTCGGGGCACTGCGCTGCAACGGCCTGCGGGCTCCCCATCCGACCCGTCTTGAAACACGGACCAAGGAGTCTGACATGCGTGCGAGTCGACGGGTTCTGAAACCTGGGATGCGCAAGGAAGCTGACGAGCGGGAGGCCCTCACGGGCCGCACCGCTGGCCGACCCTGATCTTCTGTGAAGGGTTCGAGTTGGAGCACGCCTGTCGGGACCCGAAAGATGGTGAACTATGCCTGAGCGGGGCGAAGCCAGAGGAAACTCTGGTGGAGGCTCGAAGCGATACTGACGTGCAAATCGTTCGTCTGACTTGGGTATAGGGGCGAAAGACTAATCGAACCATCTAGTAGCTGGTTCCCTCCGAAGTTTCCCTCAGGATAGCTGGAGCCCATTACGAGTTCTATCAGGTAAAGCCAATGATTAGAGGCATTGGGGACGCAACGTCCTCGACCTATTCTCAAACTTTAAATAGGTAGGATGGCGCGGCTGCTTCGGTGAGCCGTGCCATGGAATCGGGTGCTCCAAGTGGGCCATTTTTGGTAAGCAGAACTGGCGATGCGGGATGAACCGGAAGCCGGGTTACGGTGCCCAACTGCGCGCTAACCTAGAACCCACAAAGGGTGTTGGTCGATTAAGACAGCAGGACGGTGGTCATGGAAGTCGAAATCCGCTAAGGAGTGTGTAACAACTCACCTGCCGAATCAACTAGCCCCGAAAATGGATGGCGCTGAAGCGCGCGACCCACACCCGGCCATCTGGGCGAGCGCCATGCCCCGATGAGTAGGAGGGCGCGGCGGCCGCTGCAAAACCCGGGGCGCGAGCCCGGGCGGAGCGGCCGTCGGTGCAGATCTTGGTGGTAGTAGCAAATATTCAAATGAGAACTTTGAAGGCCGAAGAGGAGAAAGGTTCCATGTGAACGGCACTTGCACATGGGTAAGCCGATCCTAAGGGACGGGGTAACCCCGGCAGATAGCGCGATCACGCGCATCCCCCGAAAGGGAATCGGGTTAAGATTTCCCGAGCCGGGATGTGGCGGTTGACGGCGACGTTAGGAAGTCCGGAGACGCCGGCGGGGGCCTCGGGAAGAGTTATCTTTTCTGCTTAACGGCCTGCCAACCCTGGAAACGGTTCAGCCGGAGGTAGGGTCCAGTGGCCGGAAGAGCACCGCACGTCGCGCGGTGTCCGGTGCGCCCCCGGCGGCCCATGAAAATCCGGAGGACCGAGTACCGTTCACGCCCGGTCGTACTCATAACCGCATCAGGTCTCCAAGGTGAACAGCCTCTGGCCAATGGAACAATGTAGGCAAGGGAAGTCGGCAAAACGGATCCGTAACTTCGGGAAAAGGATTGGCTCTGAGGACTGGGCTCGGGGGTCCCGGCCCCGAACCCGTCGGCTGTCGGCGGATTGCTCGAGCTGCTCACGCGGCGAGAGCGGGTCGCCGCGTGCCGGCCGGGGGACGGACCGGGAATCGCCCCTTCGGGGGCTTTCCCCGAGCATGAAACAGTCGACTCAGAACTGGTACGGACAAGGGGAATCCGACTGTTTAATTAAAACAAAGCATTGCGATGGTCCTCGCGGATGCTGACGCAATGTGATTTCTGCCCAGTGCTCTGAATGTCAAAGTGAAGAAATTCAACCAAGCGCGGGTAAACGGCGGGAGTAACTATGACTCTCTTAAGGTAGCCAAATGCCTCGTCATCTAATTAGTGACGCGCATGAATGGATTAACGAGATTCCCACTGTCCCTGTCTACTATCCAGCGAAACCACAGCCAAGGGAACGGGCTTGGCGGAATCAGCGGGGAAAGAAGACCCTGTTGAGCTTGACTCTAGTCCGACTTTGTGAAATGACTTGAGAGGTGTAGGATAAGTGGGAGCCCTCACGGGCGCAAGTGAAATACCACTACTTTTAACGTTATTTTACTTATTCCGTGGGTCGGAAGCGGGGCATGTCCCCTCCTTTTGGCTCCAAGGCCCGGTCTTACCGGGCCGATCCGGGCGGAAGACATTGTCAGGTGGGGAGTTTGGCTGGGGCGGCACATCTGTTAAAAGATAACGCAGGTGTCCTAAGATGAGCTCAACGAGAACAGAAATCTCGTGTGGAACAAAAGGGTAAAAGCTCGTTTGATTCTGATTTCCAGTACGAATACGAACCGTGAAAGCGTGGCCTATCGATCCTTTAGATCTTCGGAGTTTGAAGCTAGAGGTGTCAGAAAAGTTACCACAGGGATAACTGGCTTGTGGCAGCCAAGCGTTCATAGCGACGTTGCTTTTTGATCCTTCGATGTCGGCTCTTCCTATCATTGTGAAGCAGAATTCACCAAGTGTTGGATTGTTCACCCACCAATAGGGAACGTGAGCTGGGTTTAGACCGTCGTGAGACAGGTTAGTTTTACCCTACTGATGACAGTGTCGCGATAGTAATTCAACCTAGTACGAGAGGAACCGTTGATTCACACAATTGGTCATCGCGCTTGGTTGAAAAGCCAGTGGCGCGAAGCTACCGTGTGCCGGATTATGACTGAACGCCTCTAAGTCAGAATCCAAGCTAGCATGCGACGCCTGCGCCCGCCGCCCGCCCCGACCCACGTTAGGGGCGCTTGCGCCCCCAAGGGCCCGTGCCATTGGCTAAGCCGGTCCGGCCGAAGTGCCGCGGCCGGCCGCCTCGAAGCTCCCTTCCCAACGGGCGGTGGGCTGAATCCTTTGCAGACGACTTAAATACGCGACGGGGCATTGTAAGTGGCAGAGTGGCCTTGCTGCCACGATCCACTGAGATCCAGCCCCATGTCGCACGGATTCGT

**Supplemental Figure S3.** **Genes at the border of the *Yr78* candidate gene region.** (a) Distal border (RefSeq v1.1. 6BS coordinate 101,735,482). Gene *TraesCS6B02G116200* coding region outside candidate region and promoter including leaf ATAC-seq peak of open chromatin inside candidate region. (b) Proximal border (RefSeq v1.1. 6BS coordinate 112,897,900). Gene *TraesCS6B02G118000* coding region and promoter outside candidate region, but 3’ region including several leaf ATAC peaks of open chromatin inside candidate region.

**Supplemental Figure S4.** **Sequence analyses supporting *TraesCS6B02G116500* and *TraesCS6B02G116600* (RefSeq v1.1) as a single pseudogene.** These two CS annotated genes (located in the – strand) are separated by a 103 bp intergenic region in RefSeq v1.1 and encode what appears to be adjacent parts of a single CC-NBS-LRR protein. However, translation is interrupted by a premature stop codon (G469*) and a 2 bp frame shift deletion. These two mutations are absent in the orthologous genes in CDC Stanley, Arina*LrFor* and CDC Landmark (H4 haplotype, Supplemental Table S10) and in the three accessions of the H5 haplotype, Supplemental Table S6). In both H4 and H5 translation can continue through the short “intergenic region” between *TraesCS6B02G116500* and *TraesCS6B02G116600* resulting in a single protein of 941 amino acids with a canonical CC-NBS-LRR structure. This protein is 83.6% similar to *Aegilops tauschii* resistant protein RGA5 (XP_020162986). Unfortunately, the incorrect annotation as two separate genes was transferred from CS to CDC Stanley, Arina*LrFor* and CDC Landmark even though the first gene lacks a stop codon:

CDC Stanley annotated as two gene

TraesSTA6B01G129900: 106423788..106425103

TraesSTA6B01G130000: 106425210..106427197

**Correct annotation:** 106423788..106427197

ArinaLrFor annotated as two gene

TraesARI6B01G133700 104036347..104037662

TraesARI6B01G133800 104037769..104039756

**Correct annotation:** 104036347..104039756

CDC Landmark annotated as two gene

TraesLDM6B01G133600: 104319332..104320647

TraesLDM6B01G133700: 104320754..104322741

**Correct annotation:** 104319332..104322741

>ArinaLrFor, CDC Stanley and CDC Landmark Complete CC-NBS-LRR protein

MTMEGIPVSAAMGALNSVLEKLATLVVDEYKLCKVMGSEIKLLTDELTFMHAFLLKMTEVEDPDVQDMVWMTAVRELSYDIEDSIDDFMQDEDAKPDGFIKKMMHMMGMWGKRKRHHQMFLDLKKRAIEVGERSERYKTRQVFSNTKNGTVDPRALAIFEHASKLVGIDEPKAELIKLLTEENGCASTQVQPKMVSIVGSGGLGKTTLANLVYQELKEKFKCKAFVSVSRNPDMTNILRTILSEVSCQGYAHTEAGSIQQLISKISDYLVDKRYFLVIDDIWDTKTWDVIKHVFPMTNCDPSLDPAQAGATCTRLPFIIITTTRMGDVACSCCLSIGGHIYNMSPLSMVHSRQLFHKRLFDSKEDLPSHLEKISEEILKKCDGLPLAIIAISSLLANKERSEQLWNQVKDSIGRALERNPSIEGMIKILSLSYFDLPSHLKTCLLYLNIFPEDSIIQKKVLIWRWIAEGFIQKDSIYSSYELGERCFNELINRSLIQPVKLNKYAKVLTCRVHDTILDFIVSKSIEENFVTFVGVPSLTIGTQSKVRRLSMQVEGEGNSIVPLSLTLYHVRSLNVFGVAVEIPSMMEFRHLRIMDFGGCHQLENHHLANVGRLFQLRYLNIRRTKVSELPEEIGCLRCLEMLDIRSTKVKDLPACIVNLQKLAHLLVNQGVRFPVGITKMQALETLKWCAWQSYIFWQELGQLKNLRKLDLNHDGAQEHKEVIASSLHNLCTQNLCSLTIWNDNDSILLNTLCTSPSLNIQKLSTRSCVLPKVPDWVGYLVNLQKLRLQVKRIRHEDLCILGALPSLLILCLENKENGENESLDRRLAVSREAGFRCLRVFTYFVEGDRMDLIFASRCMPKLEKLKTVFLGSFTDESHRSAGALYFGIENLPGLRTYKFAIRCFGTKRSNADAVKASVERLVSTHPNNRLNLIFEHGPVQS*

In CS RefSeq.v1.1 (Haplotype 2) there is a premature stop codon at G469* (yellow highlight, annotated as the end of *TraesCS6B02G116600*) and a 2 bp deletion at the Phenylalanine (F) residue 519 (red highlight, TTC to C) resulting in a reading frame shift. PI 519805 (Haplotype 1) was confirmed to be identical to RefSeq v1.1 by Sanger sequencing.

>TraesCS6B02G116600-500 in RefSeq v1.1 has a premature stop codon at G469*, and a 2 bp deletion (TT) 149 nucleotides downstream of G469*, that generates a frame shift and a second stop codon

MTMEGIPVSAAMGALNSVLEKLATLVVDEYKLCKVMGSEIKLLTDELTFMHAFLLKMTEVEDPDVQDMVWMTAVRELSYDIEDSIDDFMQDEDAKPDGFIKKMMHMMGMWGKRKRHHQMFLDLKKRAIEVGERSERYKTRQVFSNTKNGTVDPRALAIFEHASKLVGIDEPKAELIKLLTEENGCASTQVQPKMVSIVGSGGLGKTTLANLVYQELKEKFKCKAFVSVSRNPDMTNILRTILSEVSCQGYAHTEAGSIQQLISKISDYLVDKRYFLVIDDIWDTKTWDVIKHVFPMTNCDPSLDPAQAGATCTRLPFIIITTTRMGDVACSCCLSIGGHIYNMSPLSMVHSRQLFHKRLFDSKEDLPSHLEKISEEILKKCDGLPLAIIAISSLLANKERSEQLWNQVKDSIGRALERNPSIEGMIKILSLSYFDLPSHLKTCLLYLSIFPEDSIIQKKVLIWRWIAE*FIQKDSIYSSYELGERCFNELINRSLIQPVKLNKYAKVLTCRVHDTILDHSIQVH*

Avocet ‘S’ does not have the G469* premature stop codon, but has the 2 bp deletion leading to the second stop codon

>Avocet ‘S’ (Haplotype 3) TraesCS6B02G116600-500 does not have the premature stop codon G469* but has a later premature stop codon generated by the same frame shift mutation detected in RefSeq v1.1 and in PI 519805.

MTMEGIPVSAAMGALNSVLEKLATLVVDEYKLCKVMGSEIKLLTDELTFMHAFLLKMTEVEDPDVQDMVWMTAVRELSYDIEDSIDDFMQDEDAKPDGFIKKMMHMMGMWGKRKRHHQMFLDLKKRAIEVGERSERYKTRQVFSNTKNGTVDPRALAIFEHASKLVGIDEPKAELIKLLTEENGCASTQVQPKMVSIVGSGGLGKTTLANLVYQELKEKFKCKAFVSVSRNPDMTNILRTILSEVSCQGYAHTEAGSIQQLISKISDYLVDKRYFLVIDDIWDTKTWDVIKHVFPMTNCDPSLDPAQAGATCTRLPFIIITTTRMGDVACSCCLSIGGHIYNMSPLSMVHSRQLFHKRLFDSKEDLPSHLEKISEEILKKCDGLPLAIIAISSLLANKERSEQLWNQVKDSIGRALERNPSIEGMIKILSLSYFDLPSHLKTCLLYLSIFPEDSIIQKKVLIWRWIAEGFIQKDSIYSSYELGERCFNELINRSLIQPVKLNKYAKVLTCRVHDTILDHSIQVH*
